# Lipid interactions during virus entry and infection

**DOI:** 10.1111/cmi.12340

**Published:** 2014-09-11

**Authors:** Michela Mazzon, Jason Mercer

**Affiliations:** MRC-Laboratory for Molecular Cell Biology, University College LondonGower Street, London, WC1E 6BT, UK

## Abstract

For entry and infection viruses have developed numerous strategies to subjugate indispensable cellular factors and functions. Host cell lipids and cellular lipid synthesis machinery are no exception. Not only do viruses exploit existing lipid signalling and modifications for virus entry and trafficking, they also reprogram lipid synthesis, metabolism, and compartmentalization for assembly and egress. Here we review these various concepts and highlight recent progress in understanding viral interactions with host cell lipids during entry and assembly.

## Introduction

Lipids are a highly diverse group of naturally occurring hydrophobic biomolecules indispensible for cellular life. The biological functions of lipids range from membrane formation to energy storage and signalling (Voelker, 1991; van Meer *et al*., 2008; Vanhaesebroeck *et al*., 2012). As the main building block of biological membranes in eukaryotic cells, lipids serve for compartmentalization of organelles and their diverse functions. The plasma membrane, for instance, serves to selectively separate the intra- and extracellular environments and acts as the first barrier against invading pathogens (van Meer *et al*., 2008).

**Figure 1:**
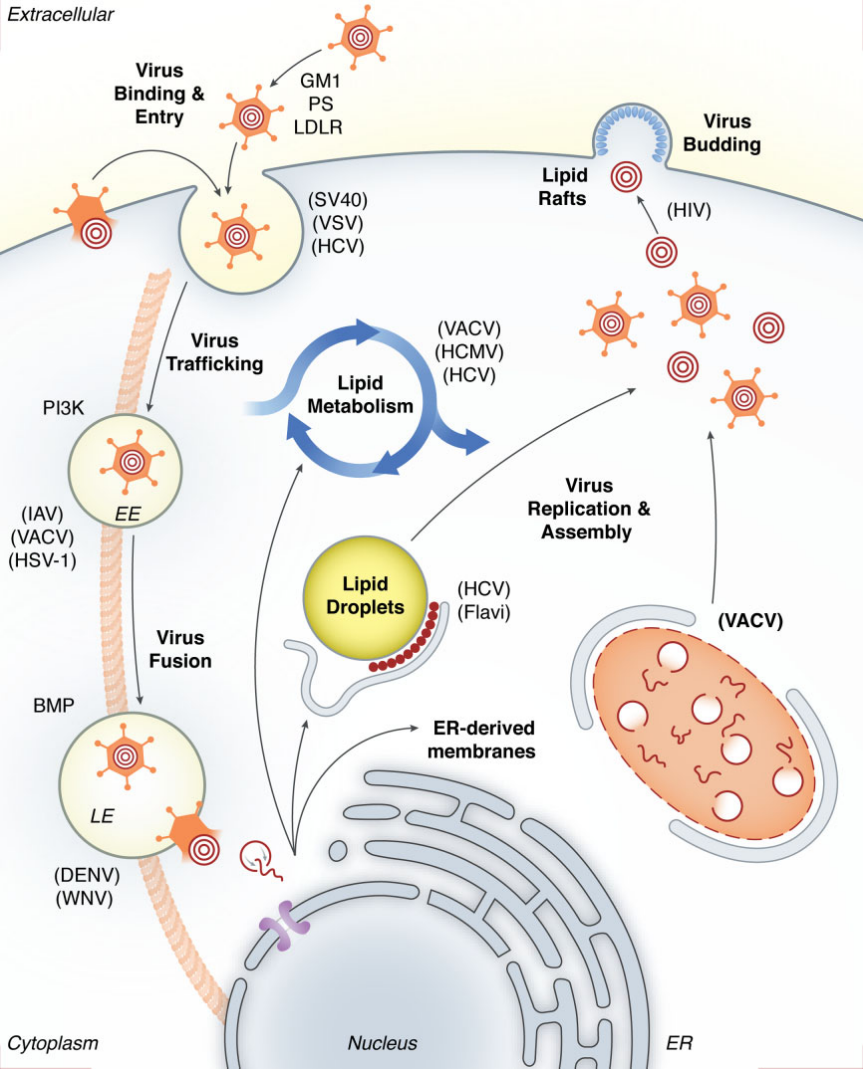
Virus-mediated subjugation and modulation of host lipids during infection. The lifecycle of most viruses proceeds through a series of basic steps: binding and internalization, fusion, uncoating of the viral genome, its replication, assembly of new particles, and budding or release of the newly made viruses. A multitude of viruses have learned to take advantage of host cell lipids during each of these stages. For binding and entry viruses hijack lipid based receptors and utilize existing lipid-based signalling. They rely on host phosphatidylinositol phosphate signalling for endosomal trafficking and differential endosomal lipid composition to assure fusion occurs at the appropriate time and place. Once viral gene expression ensues, viruses express factors to subjugate intracellular membrane compartments to build replication complexes, assemble viral factories, and alter lipid metabolism. Pictured are the stages of virus infection in which interplay between viruses and host lipids occur. The various stages in the lifecycle of a virus in which cellular lipids can be altered or subjugated are pictured with example viruses listed in parentheses (see text for details). SV40 (simian virus 40); VSV (vesicular stomatitis virus); IAV (influenza A virus); VACV (vaccinia virus); HSV-1 (herpes simplex virus 1); BMP [bis(monoacylglycero)phosphate]; DENV (dengue virus); WNV (West Nile virus); HCV (hepatitis C virus); HCMV (human cytomegalovirus); HIV (human immunodeficiency virus); EE (early endosome); LE (late endosome); GM1 (GM1 ganglioside); PS (phosphatidylserine); PI3K (phosphoinositde-3 kinase).

Investigation of the interaction between viruses and their host cells provides invaluable insights into the molecular mechanisms of viral pathogenesis and on host cell biology. How viruses enter host cells and systematically reprogram the cellular environment is one of the most compelling subjects in host–pathogen interaction. Recent work on multiple aspects of the role played by cellular lipids during the infection process has revealed their importance throughout the entire lifecycle of viruses (Heaton and Randall, 2011; Lorizate and Krausslich, 2011). These interactions range from virus binding to the host cell plasma membrane, to the release of new infectious progeny into the extracellular space. The emerging picture suggests that viruses take advantage of cell lipids by two means: subjugation and reprogramming. During early stages of infection, viruses subvert pre-existing cellular lipids and lipid signalling mechanisms for entry and trafficking. Once infection is initiated and viral genes expressed, extensive reprogramming of lipid synthesis and remodelling of lipid distribution serves to promote viral replication, assembly, and egress. This review focuses on how viruses exploit cellular lipids to promote entry and reorganize cell lipid composition, localization, and metabolism for the generation of progeny virions capable of propagating infection (illustrated in Fig. 1).

## Cellular lipids and virus entry

The first encounter between a virus and a target cell occurs at the plasma membrane. It is here that viruses first engage binding and entry receptors to initiate the infection process. In addition to acting as the main barrier between the cells and their external environment, the plasma membrane controls and co-ordinates the internalization of particles and fluids through different endocytic uptake mechanisms (reviewed in Doherty and McMahon, 2009); not surprisingly, viruses have learned to subjugate most of these for their own entry (Mercer *et al*., 2010b).

In animal cells, phospholipids account for more than half of the lipids in cellular membranes. The plasma membrane also contains glycolipids and cholesterol, the latter important for regulating membrane fluidity. While lipids mostly diffuse freely in the plasma membrane, cholesterol, glycosphingolipids (glycolipids and sphingomyelin), glycophosphatidylinositol (GPI)-anchored proteins, and transmembrane proteins can cluster into discrete domains, called lipid rafts (Simons and Ikonen, 1997; Levental *et al*., 2010; Simons and Sampaio, 2011). Lipid rafts play an important role in signalling and membrane organization, but also serve as an important platform used by both enveloped and non-enveloped viruses to enter cells (Marsh and Helenius, 2006; Mercer *et al*., 2010b; Ewers and Helenius, 2011).

### Adhesion and attachment

For most viruses, enveloped and non-enveloped, entry is preceded and enhanced by weak ionic interactions between the virion and cell surface glycosaminoglycans (e.g. heparan and chondroitin sulfate) or glycosphingolipids (Lorizate and Krausslich, 2011). These interactions promote adhesion of the virion to the cell membrane, allowing the virus to surf along the cell surface until recognition of specific binding or internalization receptors occurs. Trafficking on the cell surface or through the endosome network serves to bring the virus to its preferred site of uncoating, while providing the cues needed to activate the virus fusion machinery (Marsh and Helenius, 2006). While normally non-specific in nature, different glycosphingolipids on the viral surface have been reported to confer cell specificity: the sialyllactose moiety on human immunodeficiency virus (HIV) membrane gangliosides, for instance, has been suggested to promote uptake of HIV into mature dendritic cells (mDCs) (Izquierdo-Useros *et al*., 2012). Gangliosides are acidic glycosphingolipids carrying one or more terminal sialic acid; by using liposomes mimicking the composition and size of HIV, the authors demonstrated that gangliosides GM1, GM2 and GM3 are the key molecules that mediate liposome uptake in mDCs, with the sialyllactose moiety on gangliosides acting as a molecular recognition pattern. Recognition and uptake of sialyllactose moieties on gangliosides is likely to be a common mechanism of particle internalization by DCs, leading to antigen processing and presentation; however, in the case of HIV, the antigen-processing function of mDCs is subverted, as the virus rather uses these for virus transmission (Izquierdo-Useros *et al*., 2012).

### Lipid-mediated signalling for entry

While most viruses use surface proteins or sugars to promote entry, lipids can also function as virus entry receptors. Low-density lipoprotein receptors, negatively charged phospholipids and gangliosides have all been shown to assist entry of different viruses (Agnello *et al*., 1999; Tsai *et al*., 2003; Campanero-Rhodes *et al*., 2007; Roth and Whittaker, 2011; Izquierdo-Useros *et al*., 2012; Meisen *et al*., 2012).

Gangliosides, in particular, have been shown to be required for entry of the non-enveloped polyomavirus simian virus 40 (SV40). Upon specific binding to ganglioside GM1, SV40 reduces GM1’s diffusion rate. This stabilized SV40-GM1 complex then recruits cholesterol to generate a lipid raft. The interaction induces actin-dependent immobilization of the virus-ganglioside complex, followed by virus-induced invagination of the plasma membrane. An elegant study by Ewers *et al*. has demonstrated that association of SV40, or the isolated pentameric receptor VP1, with GM1 is sufficient to induce membrane curvatures that lead to the formation of deep invaginations in the plasma membrane of the cell (Ewers *et al*., 2010). Other polyomaviruses, as well as Shiga and cholera toxin, can also induce plasma membrane curvature and promote their endocytic uptake (Romer *et al*., 2007; Wolf *et al*., 2008).

Low-density lipoprotein receptors, also known as cholesterol receptors, are used by several members of the Flaviviridae family, including hepatitis C virus (HCV) (Agnello *et al*., 1999). The interaction is likely to occur through lipoproteins associated with the virion, such as cholesteryl esters and apolipoproteins (ApoB and ApoE) acquired during the assembly and budding of virus (Targett-Adams *et al*., 2010; Alvisi *et al*., 2011), as discussed below.

Recent reports have also indicated that the trans-membrane, cholesterol-sensing receptors Niemann-Pick C1 (NPC1) and Niemann-Pick C1-like 1 (NPC1L1) are important entry factors for filovirus (Carette *et al*., 2011) and HCV (Sainz *et al*., 2012) respectively. For filoviruses, silencing of NPC1 prevents fusion from lysosomal compartments. In contrast to NPC1L1-mediated HCV entry (Sainz *et al*., 2012), this was independent of the cholesterol binding activity of NPC1 (Carette *et al*., 2011). Whether the cholesterol transport function of NPC1 receptors is required for infectivity is unclear, but these results suggest that the NPC1 receptors can facilitate virus entry in a cholesterol-dependent and -independent fashion.

Finally, negatively charged phospholipids have been implicated as virus receptors for vesicular stomatitis virus (VSV). Entry of VSV has been suggested to require the interaction between the viral glycoprotein G and the negatively charged phospholipid phosphatidylserine (PS) on the cell surface (Carneiro *et al*., 2006). However, whether these molecules are the actual receptor for VSV entry has been challenged and is not clear (Coil and Miller, 2004).

### Lipid signalling during virus endosomal trafficking

Attachment to cellular receptors commonly activates signalling pathways that induce endocytosis of the virus. Regardless of the mechanism used for uptake, trafficking within the endosomal network is a highly co-ordinated process. After formation, endosomes go through maturation, a process that involves defined changes in cellular location, pH, protein and lipid composition (Fig. 1 for details).

The importance of lipids in co-ordinating these various events has come to light in the past decade. Phosphatidylinositol (PI) is the least abundant phospholipid in the cell membrane, but it is also one of the most versatile signalling molecules in cells and plays a central role in endosome trafficking and maturation. Differential phosphorylation of PI, regulated by specific PI kinases and phosphatases, results in the formation of different PI phosphate (PIP) species (reviewed in Vicinanza *et al*., 2008; Vanhaesebroeck *et al*., 2012). The constrained localization of these enzymes leads to differential concentrations of the PIPs within various cellular compartments; in turn, this compartmentalization influences PIPs signalling and protein recruitment activities.

Many viruses have evolved to exploit PI-mediated signalling at different stages of infection, and in particular, to co-ordinate virus entry and trigger downstream reprogramming of the host cell. The Class I PI3-kinase (PI3K) signalling pathway is one of the most important PI-mediated signalling cascades activated during virus entry. Activation of PI3K and subsequent generation of PIP3 serves as docking platform for proteins carrying lipid-binding domains, including Akt, the main effector of PI3K signalling (Das *et al*., 2010). While PI3K is involved in all forms of endocytosis (Doherty and McMahon, 2009; Mercer *et al*., 2010b; Antonescu *et al*., 2011), it is best characterized for its role in macropinocytosis, where PI3K serves to co-ordinate signalling and cytoskeletal modulation during protrusion, extension, and closure phases of macropinosome formation (Lindmo and Stenmark, 2006; Bohdanowicz and Grinstein, 2013). Activation of PI3K upon virus binding has been observed for a number of viruses using this entry mechanism, including influenza (Fujioka *et al*., 2011; Marjuki *et al*., 2011), Herpes simplex virus type 1 (HSV-1) (Zheng *et al*., 2014), HCV (Berger *et al*., 2009), Zaire Ebola Virus (ZEBOV) (Saeed *et al*., 2008), and vaccinia virus (VACV) (Mercer and Helenius, 2008; Mercer *et al*., 2010b; Izmailyan *et al*., 2012).

In addition to its crucial role in virus entry and trafficking, many viruses activate PI3K signalling for modulation of post-internalization events such as virus replication and assembly (highlighted in Diehl and Schaal, 2013). VACV, for example, requires activation of PI3K early for macropinocytic internalization (Mercer and Helenius, 2008; Mercer *et al*., 2010a; Izmailyan *et al*., 2012), and PI3K- Akt late to attenuate apoptosis of infected cells (Soares *et al*., 2009). In line with this, VACV is acutely sensitive to pharmacological inhibition of PI3K or Akt activity, which reduces viral yield up to 90% (Mercer and Helenius, 2008; Soares *et al*., 2009; Mercer *et al*., 2010a). The collective evidence indicates that activation of the PI3K signalling pathway is a widespread strategy used by multiple viruses to different ends (Diehl and Schaal, 2013).

### Lipids, membrane curvature and virus fusion

The molecular shape of membrane lipids within endosomes can strongly influence virus fusion activities. For fusion from endosomes, after internalization and activation of viral fusion machinery, the viral fusion peptide inserts into the target endosome membrane. Fusion of viral and cell membranes then proceeds through a transient hemifusion intermediate where the outer leaflet of the viral membrane mixes with the inner leaflet of the endosome membrane. Subsequently, the inner viral and outer endosomal membrane leaflets merge and the hemifusion stalk opens, forming the fusion pore and thus completing the fusion process (Lorizate and Krausslich, 2011). Interestingly, lipid composition influences from which endosomal compartment a virus will fuse. For instance, dengue virus (DENV) exploits the late endosome specific anionic lipid bis(monoacylglycero)phosphate (BMP) to promote fusion from late endosomes. The lipid-dependence of DENV fusion machinery assures that DENV does not fuse prematurely (Zaitseva *et al*., 2010). Endosomal lipid content has also been shown to be important for West Nile and alphavirus fusion, requiring either cholesterol or both cholesterol and sphingolipids respectively (Kielian *et al*., 2010; Moesker *et al*., 2010). Given the importance of lipids during this stage of virus entry, it comes to no surprise that host cells have developed strategies to counteract the interaction between viral and cellular membranes. Recent description of interferon-inducible transmembrane (IFITM) proteins and 25-hydroxycholesterol as viral restrictions factors serve as good examples (see Appendix 1).

## Cellular lipids during viral infection

Subjugation of cellular lipids by viruses is not only used to promote entry and intracellular trafficking. In fact viruses also use lipids to modify their own proteins as well as cellular factors to promote viral replication complex formation, production of new viral particles, viral egress and spread of infection.

### Lipids and viral replication complex formation

Viruses that replicate in the cytosol tend to reorganize cellular membranes to create sites of active replication. These sites, called viral factories or replication complexes (RC) provide a scaffold for the viral replication machinery, serve to concentrate the viral and cellular factors needed for assembly of new virions, and provide a protective environment for avoidance of cellular innate immune responses. Examples include the large dsDNA poxviruses which transiently recruit endoplasmic reticulum (ER)-derived cisternae around viral RCs (Condit *et al*., 2006; Krijnse Locker *et al*., 2013), and positive stranded RNA viruses which reorganize ER, Golgi, endosomal, lysosomal or mitochondrial membranes to form specialized membrane-bound RCs (Miyanari *et al*., 2007; Miller and Krijnse-Locker, 2008; Welsch *et al*., 2009; den Boon *et al*., 2010; Heaton *et al*., 2010).

A number of studies are now shedding light on virus-mediated modification of lipid profiles in order to shape the cellular membrane environment to promote infection. Membrane remodelling induced by DENV, for instance, is directly linked to a shift in the lipid repertoire during infection (Perera *et al*., 2012). High-resolution mass-spectrometry studies in mosquito cells indicates that together with lipids involved in controlling membrane fusion, fission, trafficking and cytoskeletal function, those able to change membrane curvature or permeability are enriched in DENV infected cells (Heaton *et al*., 2010; Perera *et al*., 2012).

Recent work has revealed that several of these viruses co-opt cellular cofactors to facilitate virus-mediated membrane remodelling. The NS5A protein of Hepatitis C virus (HCV), for instance, recruits PI4K-IIIα to virus replication sites to increase local levels of PI(4)P (Alvisi *et al*., 2011), while the picornavirus protein 3A recruits PI4K-IIIβ to the same end (Greninger *et al*., 2012). It has been proposed that high levels of PI(4)P directly contribute to RC formation by influencing membrane bending or by regulation of intracellular processes including vesicle fusion, budding and sorting (Berger *et al*., 2009; Alvisi *et al*., 2011).

In addition to subverting lipids for its replication, HCV subjugates existing lipid droplets (LDs) for assembly of infectious viral particles (Miyanari *et al*., 2007). Under normal conditions, lipid droplets serve as storage organelles for neutral lipids. During HCV infection viral core protein associates and accumulates on LDs. These virus-modified structures become surrounded by HCV RC-containing membranes and associated viral RNA and non-structural proteins. Although not fully understood, co-ordinated events occur between HCV RCs containing replicated genomes, and the viral core containing LDs for complete genome packaging and virus assembly (Miyanari *et al*., 2007).

### Virus subversion of lipid metabolism

That viruses impact cell metabolism has been known for many years. However, advances in the field of metabolomics have only recently allowed us to fully appreciate the extent to which viruses reprogram host cell metabolism. Recent studies on several different viruses indicate that metabolic pathways and enzymes tend to be manipulated in a virus specific fashion. This is likely dependent upon the metabolic precursors the individual viruses find most advantageous for their replication. Not too surprisingly, all of these studies have identified important alterations in lipid synthesis upon viral infection. By altering the activity of the tricarboxylic acid (TCA) cycle or by manipulating the carbon source used by the cells to generate energy and macromolecules, several viruses take control of central energy metabolism to promote synthesis of cholesterol and fatty acids. This phenomenon has been described for two large enveloped viruses: HCMV (Vastag *et al*., 2011; Yu *et al*., 2011) and VACV (Greseth and Traktman, 2014). This is likely to be required to assure sufficient viral lipid membrane for building new viral progeny, and for remodelling of viral and cellular membranes to enhance viral replication, egress and entry into neighbouring cells.

Large enveloped viruses are not the only viruses that manipulate cellular lipid metabolism. Microarray analysis of HCV infected cells show significant changes in the expression of genes involved in lipid metabolism (Woodhouse *et al*., 2010). Recent transcriptomic and proteomic analyses indicate that the expression of host genes involved in lipid biosynthesis, degradation, and transport is profoundly altered by HCV; in particular cholesterol biosynthesis genes were found to be upregulated (Woodhouse *et al*., 2010). Parallel lipidomics analysis also showed changes in selected lipid species, particularly phospholipids and sphingomyelin (Diamond *et al*., 2010). This suggests that HCV reprogramming of host lipid metabolism attempts to maintain host homeostasis in spite of the elevated demand of metabolic precursors by the virus.

## Modulation of cell lipids for virus assembly and infectivity

To complete the cycle of infection, newly assembled virions must have the ability to infect naive cells. Thus the importance of lipids during this last stage of the viral life cycle is twofold: not only are they required for assembly and release of new viral particles, in some cases the lipid content of the viral particles dictates virus infectivity.

Most enveloped viruses acquire their envelope by budding. This can occur either at the plasma membrane or internal membranes. The majority of retroviruses, paramyxoviruses, and orthomyxoviruses bud from the plasma membrane; flaviviruses bud from the ER membrane, and HSV gains its envelope from trans Golgi network (TGN) or endosomes (Lorizate and Krausslich, 2011). As lipids play a central role in this process, it is not surprising that viruses promote lipid synthesis and reorganization of cellular membrane lipid composition for virus assembly and exit. HIV, which buds from plasma membrane lipid rafts provides an interesting example. HIV actively modulates lipid rafts by increasing the synthesis and trafficking of cholesterol to these sites (Aloia *et al*., 1993). Analysis of the viral membrane has revealed that it is enriched in sphingolipids and cholesterol at the expense of phosphatidylcholines, confirming its raft origin (Aloia *et al*., 1993). Also, the major HIV structural protein responsible for assembly of the budding structure, Gag, localizes in lipid rafts through interactions with the lipid signalling molecule PIP2 to ensures the correct localization of Gag for viral assembly and budding (Aloia *et al*., 1993; Barrero-Villar *et al*., 2008; Lorizate *et al*., 2013).

In addition to modifying the lipid content of existing membranes to co-ordinate virus budding, viruses can promote the active synthesis of lipids to provide membranes for the viral envelope. The increased biosynthesis of fatty acids, observed during HCMV and VACV infections is thought to increase the available membranes for viral membrane wrapping and assembly respectively. Interestingly in these cases the viral lipid content is altered with respect to host cell membranes. HCMV has been shown to contain more phosphatidylethanolamines and less phosphatidylserine than the host cell membrane. This lipid content, which resembles that of synaptic vesicles, is thought to facilitate HCMV budding and release (Liu *et al*., 2011).

In contrast, the VACV membrane is enriched for phosphatidylserine (PS) (Ichihashi and Oie, 1983) facilitating the next round infection by PS-dependent macropinocytic entry (Mercer and Helenius, 2008). With the exposure of PS, VACV mature viruses (MVs) resemble apoptotic bodies. As apoptotic clearance can occur by macropinocytosis (Hoffmann *et al*., 2001) we postulated that VACV employs apoptotic mimicry (Mercer and Helenius, 2010). Despite our demonstration that the virus can directly bind to the PS receptor Axl (Frei *et al*., 2012), the PS receptors (TAMs, TIMs, stablin-2, MFGE-8) and bridging molecules (serum protein S and Gas6) (Scott *et al*., 2001; Hanayama *et al*., 2002; Hafizi and Dahlback, 2006; Miyanishi *et al*., 2007; Park *et al*., 2008) used by VACV remain elusive. It will be of interest to determine if VACV PS receptor(s) bind phosphatidylglycerol or the D-stereoisomer of PS, two lipids absent from the MV membrane that can functionally substitute for the naturally occurring PS (Laliberte and Moss, 2009).

Since its inception, viral apoptotic mimicry has proven to be a widespread lipid-mediated entry mechanism used by several enveloped viruses including: pichinde, cytomegalo, lassa fever, lenti, dengue, ebola and Marburg viruses (Callahan *et al*., 2003; Shimojima *et al*., 2006; Soares *et al*., 2008; Hunt *et al*., 2011; Kondratowicz *et al*., 2011; Mercer, 2011; Meertens *et al*., 2012; Jemielity *et al*., 2013; Moller-Tank *et al*., 2013; Morizono and Chen, 2014). For several of these viruses the PS-receptors and bridging molecules required for entry have now been defined (Shimojima *et al*., 2006; Hunt *et al*., 2011; Kondratowicz *et al*., 2011; Mercer, 2011; Meertens *et al*., 2012; Jemielity *et al*., 2013; Moller-Tank *et al*., 2013). That antibodies directed against PS can neutralize lethal pichinde and cytomegalo virus infections (Soares *et al*., 2008), and virus engagement of PS receptors can dampen innate immune responses to infection (Bhattacharyya *et al*., 2013) suggests that the therapeutic potential of PS targeting deserves further investigation.

## Future perspectives

The fundamental role of lipids in virus biology and infection is becoming increasingly clear. New technologies, such metabolomics, and advances in mass spectrometry-based lipidomics are allowing for systematic characterization of the alterations in host lipid metabolism, as well as cellular and viral lipid profiles induced by viral infection.

As highlighted in this review, lipids serve to orchestrate different stages of viral replication, ranging from entry to spread. Viruses take control of lipid-mediated signalling to co-ordinate viral entry and intracellular trafficking. Later during infection, they actively modify intracellular membrane for replication, re-direct lipid metabolism to produce sufficient membrane for the assembly of new particles, and modify cell membrane lipid content to ensure infectivity of those virions.

Systematic characterization of how viruses take control of and alter lipid metabolism is now needed to unravel the common strategies used by these different viruses. Such studies may serve for the identification of infection biomarkers; and together with the development of therapeutics targeting subsets of lipid synthesis enzymes it may be possible to identify novel broad-spectrum therapeutic agents that target virus modified lipid metabolism. Overall, a deeper understanding of how viruses manipulate the host cells lipid program will serve to further our understanding of the cellular mechanisms that govern lipid modification, compartmentalization and metabolism.

## Appendix 1

### IFITMs and 25-HC as viral restriction factors

About 20 years after their identification as interferon stimulated genes (ISGs) (Lewin *et al*., 1991), interferon-inducible transmembrane (IFITM) proteins were characterized as viral restriction factors (Brass *et al*., 2009). Interestingly, while most known restriction factors act after viral entry, IFITM proteins were shown to restrict viral fusion. Since the initial study describing the role of IFITM proteins as influenza A virus (IAV) and flavivirus restriction factors (Brass *et al*., 2009), several other studies have followed showing the importance of these proteins in inhibiting entry of many other viruses including filoviruses (Huang *et al*., 2011), rhabdoviruses (Weidner *et al*., 2010; Smith *et al*., 2013), bunyaviruses (Mudhasani *et al*., 2013), and the non-enveloped reoviruses (Anafu *et al*., 2013).

Each of the three IFITM proteins has different cellular localizations which determine the range of viruses they restrict. IFITM1 is located primarily at the plasma membrane; IFITM2 and 3 localize in intracellular compartments, with IFITM3 colocalizing with endosomal markers (reviewed in Smith *et al*., 2014). IFITM3 has been shown to block fusion of viral and cell membranes – plasma or endosomal – via reduction of membrane fluidity (Li *et al*., 2013). While the exact mechanism of action is not clear, one study suggests that IFITM3 reduces membrane fluidity and increase positive curvature in the outer leaflet of the host membrane: by interacting with vesicles membrane protein associated protein A (VAPA), IFITM3 would disrupt the association between VAPA and an oxysterol binding protein that regulates the content of cholesterol in endosomal membrane, therefore restricting viral fusion (Amini-Bavil-Olyaee *et al*., 2013). More recent reports have however contradicted this hypothesis, demonstrating that an increase in the concentrations of cholesterol in cellular membranes is not itself sufficient to inhibit fusion, and suggests instead that, directly or indirectly, IFITM3 might interfere with pore formation in the cytoplasmic leaflet of the hemifusion intermediate or, alternatively, that they might re-direct viral fusion to a non-productive pathway (Desai *et al*., 2014). Inhibition of non-enveloped viruses as reovirus, and lack of inhibition of enveloped viruses like arenaviruses (Brass *et al*., 2009) and some major DNA viruses like papilloma virus, human cytomegalovirus and adenovirus (Warren *et al*., 2014) also suggest that much more still needs to be discovered, and also introduces the possibility that some viruses might have evolved to specifically evade this antiviral mechanism.

Recent findings also indicate that repression of the sterol biosynthetic pathway is an important component of the interferon-inducible antiviral response (Blanc *et al*., 2011). An overexpression screen aimed at identifying IFN-induced antiviral effectors has clarified a role for 25-hydroxycholesterol (25HC) in inhibiting infection of several enveloped viruses (Liu *et al*., 2012). 25HC was reported to inhibit virus-cell fusion through direct alteration of cellular membrane composition (Liu *et al*., 2013), although it is also possible that viral infection is modulated at multiple post-entry events (Schoggins and Randall, 2013).
